# A systematic review of poeciliid fish invasions in Africa

**DOI:** 10.1186/s12862-024-02321-3

**Published:** 2024-11-06

**Authors:** Joshua Pritchard Cairns, Pedro Henrique Negreiros de Bragança, Josie South

**Affiliations:** 1 Water@Leeds, School of Biology, Faculty of Biological Sciences, University of Leeds, Leeds, LS2 9JT UK; 2 South African Institute for Aquatic Biodiversity, Private Bag 1015, Makhanda, Eastern Cape 6140 South Africa; 3 Department of Ichthyology, American Museum of Natural History, New York, NY 10024-5102 USA

**Keywords:** Aquarium, Biocontrol, Ecological impact, *Gambusia*, Introduction pathway, Invasive species, Ornamental trade

## Abstract

**Background:**

This review compiles and synthesises the existing information concerning non-native poeciliid introductions to Africa. The recent upsurge in research on invasive poeciliids has revealed their widespread occurrence in Africa.

**Results:**

Within the 87 relevant articles, 74% reported on the presence of *Gambusia* spp., 33% on *P. reticulata*, 19% on *X. hellerii*, 11% on *X. maculatus*, and 5% on other ornamental poeciliids. Overall, poeciliids have been documented as introduced to 25 different countries in Africa. With *Gambusia* spp. being introduced to 16 countries and *P. reticulata* to 19 countries. Our results are representative of the current state of research on invasive poeciliids in Africa. There was a concentration of studies in South Africa, with limited research elsewhere. Current distribution data is relatively patchy, although widespread surveys of multiple river systems in Morocco and South Africa, confirmed widespread and abundant established poeciliid populations. The ecological impacts of invasive poeciliids in Africa remain understudied but evidence indicates deleterious effects on native fish, invertebrates, and amphibians, many of which are critically endangered or endemic.

**Conclusion:**

Current research is limited in reporting from certain countries and ecological impacts. An increased effort to monitor species composition in vulnerable waterbodies, especially in the many African countries where invasive poeciliids are reported, should be completed to reveal further established populations. Future research should prioritise quantifying the ecological impacts of invasive poeciliids in the field and identifying both vulnerable and resistant native ecosystems to guide future management decisions.

**Supplementary Information:**

The online version contains supplementary material available at 10.1186/s12862-024-02321-3.

## Background

Non-native species that have established populations beyond their natural range and are causing ecological impact are considered invasive [13]. Globalisation and international trade are redistributing non-native species and increasing introduction rates [161], whilst climate change is acting synergistically to shift species ranges, exacerbate their environmental impacts and complicate management efforts [86]. Successful invaders often exploit niche space in native ecosystems and establish viable populations directly impacting native species and the system [3]. Introductions can increase interspecific competition [84], predation pressure [25], transmit novel diseases [38] and co-introduced parasites [115], all of which contribute to the population declines of native and endemic species. Changes to community composition can alter food webs and nutrient transfer pathways [43], reduce genetic and functional diversity [148], and affect the physical characteristics of habitats through ecosystem engineering [80]. Invasive species may also facilitate the establishment of other introduced species through “invasion meltdown” processes [166]; these impacts can propagate across other trophic levels [73] and contribute to an overall decline in biodiversity and biotic resistance [183]. The combination of invasive species and other drivers have contributed to 60% of all global extinctions [12, 95]. Furthermore, the economic burden of invasive species is accelerating and quadrupling every decade [49].

Human populations are also beneficiaries from the services provided by freshwater ecosystems [79, 188], yet such key environments have been overlooked in conservation targets until recently (30% by 2030 [27, 41]). Freshwaters exhibit disproportionate species richness per unit area and are subject to high levels of human exploitation, becoming highly vulnerable to anthropogenic and environmental stressors, including species invasion [52, 176]. The threat of invasive species to freshwaters occurs concomitantly with other threats emanating from human resource exploitation, and negative impacts are likely synergistic. These include pollution, disturbance, and alteration of hydrological flows, which are underpinned by the environmental drivers of climate change, nitrogen deposition, and changes to the water cycle [52]. Furthermore, the transport mechanisms relocating freshwater species are advancing, including improved trade routes and the threat of unregulated e-commerce [150]. Thus, freshwaters are experiencing widespread species declines and losses in biodiversity [24] and the introduction of non-native species is a key factor driving these impacts [153].

Poeciliidae is a family of freshwater fish comprising 274 valid species [70], many of which are popular in the ornamental aquarium trade due to ease of culture and broad tolerance to environmental conditions. Their native distribution range lies within the American continent, extending across locations from the Atlantic to the Pacific coasts. The northernmost records of naturally occurring poeciliids are in the southern United States, while the southernmost records are in the Argentinean Pampas (pers comms – P. Bragança). Poeciliids have been introduced to all continents other than Antarctica [64] and are easily recognized by the presence of the gonopodium in males, an intromittent organ formed by modified anal fin rays 3,4 and 5, and viviparity or ovoviviparity among females.

Poeciliids exhibit life history and behavioural traits favoured in aquaculture, which are related to invasion success [11]. For example, their short generation times [18] female-dominated populations and early maturing males [169] enable their proliferation across a wide salinity gradient [32, 134]. When introduced to isolated habitats, even a single pregnant female can establish a population [26, 45]. Some species can even produce viable populations in hypoxic and polluted environments [159, 177] and adapt rapidly in response to biotic pressures such as predator abundance [108], prey availability [123], and changing habitat characteristics [87]. Combining their reproductive mechanisms with high dispersal tendencies [51], aggressive behaviour [64] and polyphagous feeding habits [54], invasive poeciliids can quickly colonise novel ecosystems.

Invasive poeciliids can have detrimental impacts on native biota and ecosystem functioning. They have caused native species population declines at multiple trophic levels: through the co-introduction of alien parasites [63, 69]; predation of invertebrates [165], amphibians [164] and small fish [160]; hybridisation with closely related native species [64]; as well as negative interactions from interference competition [23] and aggressive behaviour [180]. Impacts from established invasive poeciliids can restructure native communities [90] and influence local environmental conditions [92]. Poeciliids have been primarily cultured and translocated to new continents through mosquito biocontrol programmes [123] and the international pet trade pathways [64]. Therefore, both intentional and accidental aquaria release are thought to be the predominant introduction vector [140].

Given that Africa is a malaria hotspot there have been many poeciliid biocontrol introductions into the continent in the past century. Despite the threat that poeciliid invasions represent to native ecosystems, there is still limited and patchy information regarding the distribution, spread and impacts of non-native poeciliids. Another main knowledge gap in Africa is related to its native freshwater taxa, especially its fish diversity. At the same time, the freshwater ecoregions of Africa are extremely diverse, displaying high levels of endemism and consisting of several biodiversity hotspots, yet there are few taxonomy experts in the continent or working with African fish fauna [42]. This scenario is referred to as taxonomic impediment, which is a major challenge in delimiting and estimating the continent’s freshwater fish diversity, and consequently in estimating the impact of invasive poeciliids [22, 60, 78, 122, 168]. Furthermore, there are many functionally analogous endangered and endemic fish species (e.g. Aphaniidae and Procatopodidae) which may be threatened directly by poeciliid invasion impacts [74, 162], whilst threat from invasion to the cryptic freshwater biodiversity in Africa is unknown.

The purpose of this review is to collate and analyse the available information relating to poeciliid introductions into African countries. Where possible, a summary is provided on the introduction events, establishment success, dispersal, and impacts. By evaluating the available literature, we can improve our understanding of the distribution of invasive poeciliids in Africa and the consequences of their introductions. Assessment of the results will allow the recognition of recurring pathways, vulnerable freshwater systems and significant gaps in knowledge. Based on our findings, native ecosystems and species requiring urgent ecological investigation can be identified and poeciliid management strategies can be informed.

## Methods

This study followed the updated PRISMA 2020 guidelines for conducting and reporting systematic reviews [141]. Relevant literature was identified by specifying inclusion criteria, search strategies, exclusion methods, and outcomes for the required data. The search terms “poeciliid” AND “invasive” AND “Africa” were searched in the Google Scholar search engine. This initial search produced limited results, possibly due to the infrequent use of the term “poeciliid”. As such, further searches were undertaken replacing “poeciliid” with the names of the eight introduced species, preliminarily identified by scanning initial search results. The results retrieved were dated up to March 2023 and assessment completed by one reviewer independently. Articles considered for inclusion must have provided information on introduced poeciliids in Africa, encompassing any details of the following: year of introduction, origin, vectors, invasive pathways, specific locations, establishment success, persistence, dispersal activity, impacts on native biota and ecosystems. The identification of relevant literature was conducted in a step-by-step exclusion process. First the title of each article generated was considered and those with evidently unrelated content were excluded. The abstracts of the remaining articles with potentially relevant titles were then examined. Relevant abstracts were then carefully reviewed, and it was determined whether they fit at least one of the inclusion criteria. After consideration of the full text, articles containing relevant information were compiled into a database. Reference lists were examined, and additional relevant articles were obtained applying the same exclusion procedure. To achieve comprehensive results, a recursive citation search (“snowballing”) was undertaken to find additional studies. Where articles were not accessible, the South African Institute for Aquatic Biodiversity (SAIAB) library platform was used to gain access to copies, or in some cases authors were contacted directly to request access. Expert insights via personal communications, and consultation of the Global Biodiversity Information Facility (GBIF) supplemented the findings. Since the GBIF databases include numerous iNaturalist records, the iNaturalist database was also directly searched for occurrences (iNaturalist community; S1). For both databases, separate searches were conducted for each poeciliid species, applying “Africa” as the continent filter. Following compilation of the material sourced, the findings were categorised according to each different poeciliid species recorded as having been introduced to the African continent.

To synthesise ecological impact, each publication was scrutinised to complete an Ecological Impact Categorisation of Alien Taxa (EICAT) [85] assessment whereupon country, poeciliid species, impacted taxa, mechanism of impact and confidence of impact evidence were recorded along with justification for scoring (S2, S3).

## Results

**Fig. 1:**
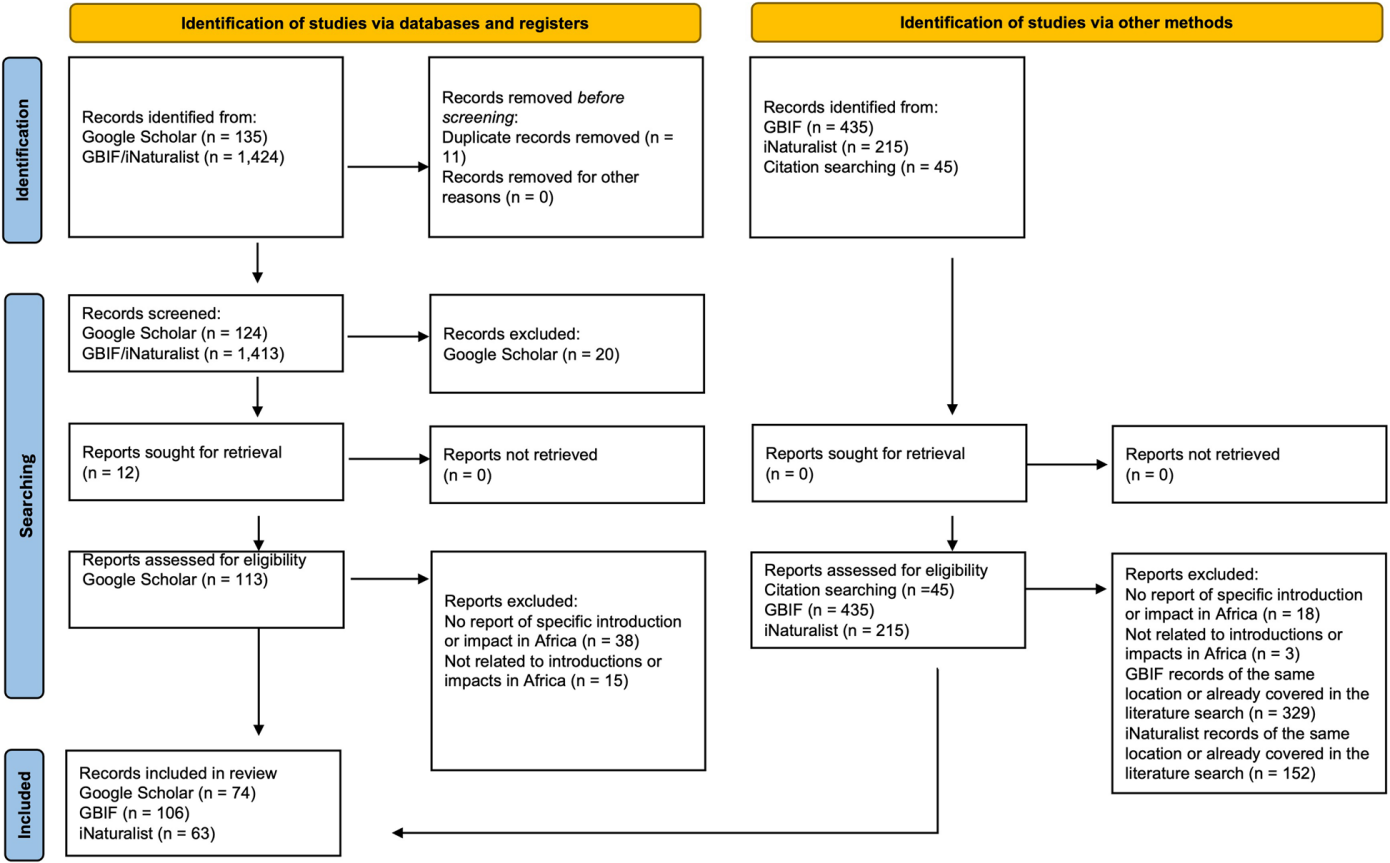
PRISMA flow diagram showing the process for the identification, screening, and inclusion of relevant articles and citizen science platforms (n = number of articles) [82]. All underlying data is in S1

The searches undertaken produced the results that follow. First, the term “poeciliid” retrieved 426 results of which nine were relevant. Replacing “poeciliid” with the species names produced the following results by species with the number of additional articles matching the inclusion criteria shown in brackets: “*Poecilia reticulata*” retrieved 1,500 results (16), “*Gambusia affinis*” retrieved 1,570 results (36), “*Gambusia holbrooki*” retrieved 1,090 results (3), “*Xiphophorus hellerii*” retrieved 190 results (4), “*Xiphophorus maculatus*” retrieved 227 results (1), “*Poecilia latipinna*” retrieved 269 results (2), “*Poecilia sphenops*” retrieved 175 results (0), “*Poecilia velifera*” retrieved 53 results (0) (Fig. 1). There was a single report of *Phalloceros* spp. in the literature, however this was excluded as a search term because although the specimen was confirmed as the genus *Phalloceros*, the species was not known as there was only one *Phalloceros* species described at the time of recording (1976; Jubb et al. [98]), but 20 new species were described in 2007. The reference search produced three relevant French articles and so an additional search was carried out using the terms “poeciliidés” AND “envahissant” AND “Afrique” but retrieved no relevant results. The subsequent replacement of “envahissant” with “introduction” produced nine results, although the only relevant articles retrieved were previously included from the reference search. Eight of the total results were categorised as grey literature; however, since they were all sourced from the reference search of peer-reviewed articles, their information was deemed reliable (Fig. 1). From the search results, a key article on *G. holbrooki* introductions was published on the Global Biodiversity Information Facility (GBIF) website (GBIF.org [75], S1), which contributed 62 occurrences [173]. The total number of unique results from GBIF/iNaturalist reports, excluding duplicates from the Google Scholar search and repeated GBIF/iNaturalist records, were as follows: *G. affinis* (36), *G. holbrooki* (16), *P. reticulata* (81), *X. hellerii* (23), *X. maculatus* (13) (S1).

**Fig. 2:**
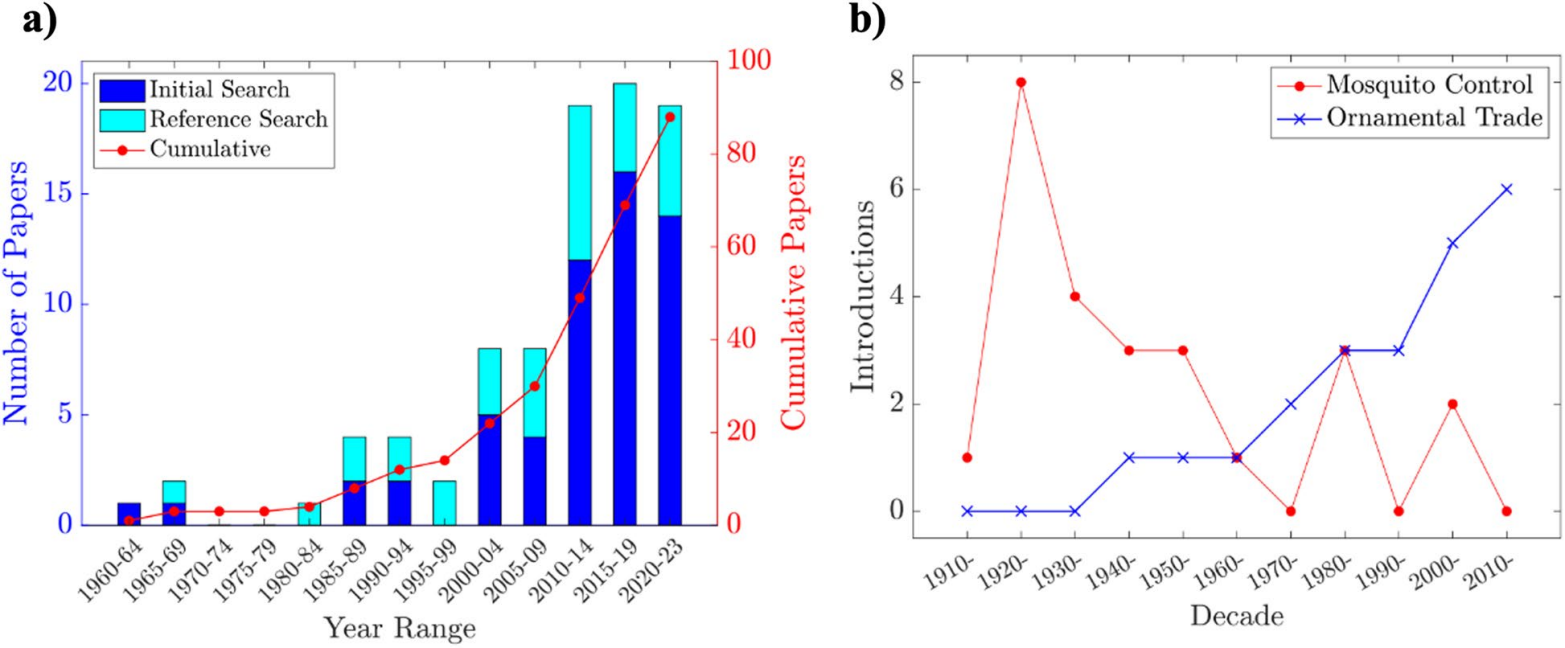
**a** Number of relevant articles published each 5 years from 1960 to 2023, **b** Number of poeciliid introductions each decade from mosquito biocontrol and the ornamental trade

The introduction of nine different poeciliid species have been confirmed across 25 African countries: the guppy, *Poecilia reticulata* Peters, 1859, the western mosquitofish, *Gambusia affinis* Baird and Girard, 1854, the eastern mosquitofish *Gambusia holbrooki* Girard, 1859, the green swordtail, *Xiphophorus hellerii* Heckel 1848, the southern platyfish, *Xiphophorus maculatus* Günther, 1866, the sailfin molly, *Poecilia latipinna* Leseur, 1821, the common molly, *Poecilia sphenops* Valenciennes, 1846, the Yucatan molly, *Poecilia velifera* Regan, 1914, and an undetermined species of the genus *Phalloceros*. Articles identified as relevant to poecillid introductions in Africa date from 1962 to 2023. Publication frequency increased from the 1980s onwards with the greatest number of articles published from the start of 2015 to the end of 2019 (Fig. 2a). Of the 88 included articles, 74% reported on introductions of *Gambusia* spp., 33% on *P. reticulata*, 19% on *X. hellerii*, 11% on *X. maculatus*, and 5% attributed to the remaining species. Some articles provided information on multiple species. All underlying data on distribution, occurrence, vector, establishment status, and impact, including GBIF and iNaturalist information, is summarised in S1a.

*Gambusia affinis* and *Gambusia holbrooki* were introduced primarily for vector mosquito biocontrol, while *Poecilia reticulata* was introduced for both vector mosquito biocontrol and by the ornamental pet trade. The remainder of the species are popular ornamental fish. The primary causes of poeciliid invasions in Africa are vector mosquito biocontrol and the ornamental fish trade. Biocontrol-related introductions predominated the first half of the 20th century, whereas invasive populations associated with the ornamental trade have become increasingly frequent since the 1950s (Fig. 2b).

### *Gambusia* spp.

*Gambusia* spp. were reported in 39% of African countries (S1). Results for *G. affinis* and *G. holbrooki* are combined because both were considered subspecies of *Gambusia affinis* until approximately 1990 [185]. Nonetheless, literature descriptions of the individual species are acknowledged, even in cases of articles before 1990.

**Fig. 3:**
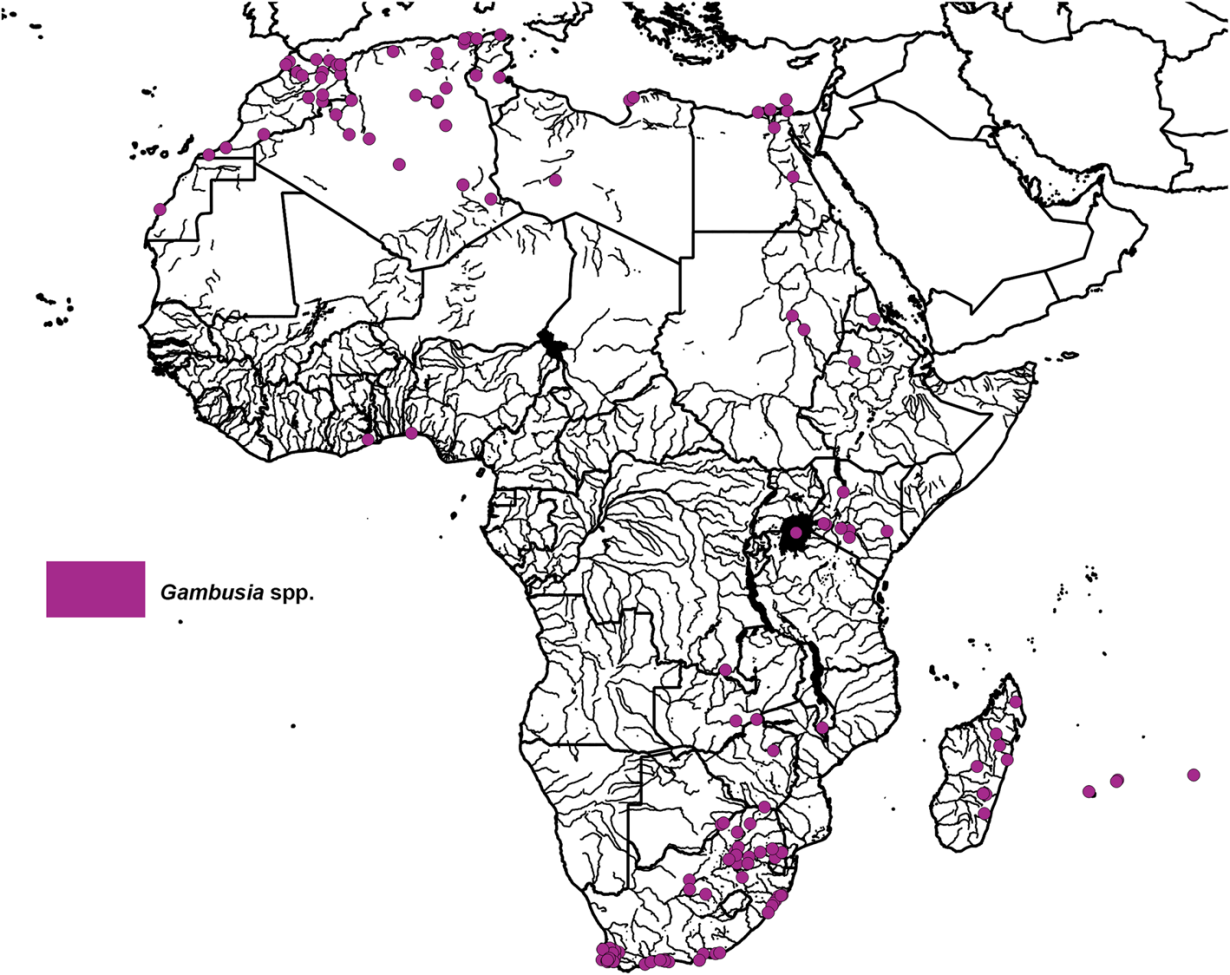
Distribution map of the reported locations for Gambusia spp. in African freshwater systems. All underlying data and sources reported in S1

*Gambusia* spp. are reported as widespread and established throughout Northern Africa. During the 1920s and 1930s, *Gambusia* spp. were introduced to Egypt and Algeria from North America [111, 185]; to Egypt and Libya from Italy [96, 185]; and to Morocco by French colonists [7] (Fig. 3; S1). Of these introductions, only the *G. holbrooki* introduced to Algeria were intended for mosquito biocontrol [111]. Established *G. holbrooki* populations are widespread and abundant in the north-east of Morocco [173]; this species is the most frequently reported alien species in Moroccan freshwaters [174]. *Gambusia holbrooki* is also considered to be common throughout Algerian wetlands [100] and has been reported at a range of freshwater habitats including irrigation ditches, dune ponds, and dams [10, 29, 163]. *Gambusia affinis* populations are established in Egypt, Libya, and Tunisia and were recently reported in freshwater habitats such as canals [171], lakes (Shaltout et al., 2016); [61], oases [170], and a coastal lagoon [104].

Reports of *Gambusia* spp. in Western and Central Africa are sparse (Fig. 3; S1). *Gambusia affinis* was reported in polluted sewers in Lagos, Nigeria [46] and is understood to be common in Ghanaian freshwater systems [139] but has only been reported at one location [14]. Multiple specimens were collected in freshwater habitats in the Democratic Republic of the Congo during 1946-47 and in 1963 [170], but there is no information on their current establishment.

*Gambusia* spp. were introduced to multiple countries in Eastern Africa for mosquito control (Fig. 3; S1). In the 1920s, *Gambusia affinis* were introduced to Madagascar and Zimbabwe from the United States [111, 185] and to South Sudan from Italy [186]. *Gambusia* spp. were translocated from within Africa via introductions to South Sudan from Egypt in the 1930s [186] and Zambia from South Africa in the 1940s [5]. *Gambusia* spp. have established in artificial habitats in Zimbabwe [111], established in urban waters in Sudan [58], sampled in several freshwater ecosystems in Madagascar in the 2000s [113, 184], and are harvested from rivers and resold for private mosquito control in Zambia (Pers comms – A. Jere). *Gambusia affinis* was also introduced to Kenya for mosquito biocontrol [66], although the date of introduction is unknown, and was first reported in the 1960s [120]. *Gambusia affinis* populations are now considered established and widespread throughout Kenyan river basins [135]. Furthermore, *Gambusia* spp. were introduced to Comoro Islands [185], although the date is unknown, and were reported in Eritrea, Malawi, and the Mascarene Islands [34, 71]. *Gambusia affinis* was introduced South Africa in 1936 from North America for mosquito control [44] and naturalised populations were first sampled in the 1960s [34] and then were reported in multiple locations in the 1980s in both the Western Cape and Eastern Cape [44] (Fig. 3; S1). At the beginning of the 21st century, *G. affinis* populations were established in 50% of South African river systems [143].

Recent studies have documented abundant and widespread populations of *G. affinis* in KwaZulu-Natal, Western Cape, and Eastern Cape provinces [35, 65, 190] (Fig. 3; S1). Occurrence recordings have continued to detect *G. affinis* in these provinces during the last 5 years, with additional scattered records from the Northern Cape, Limpopo, Mpumalanga, and Gauteng [93, 99]; S1). Additionally, *G. affinis* were recently reported near the South African border in Botswana [99] and were reported in the Hhohho Region of Eswatini [93]; S1.

### *Poecilia reticulata*

**Fig. 4:**
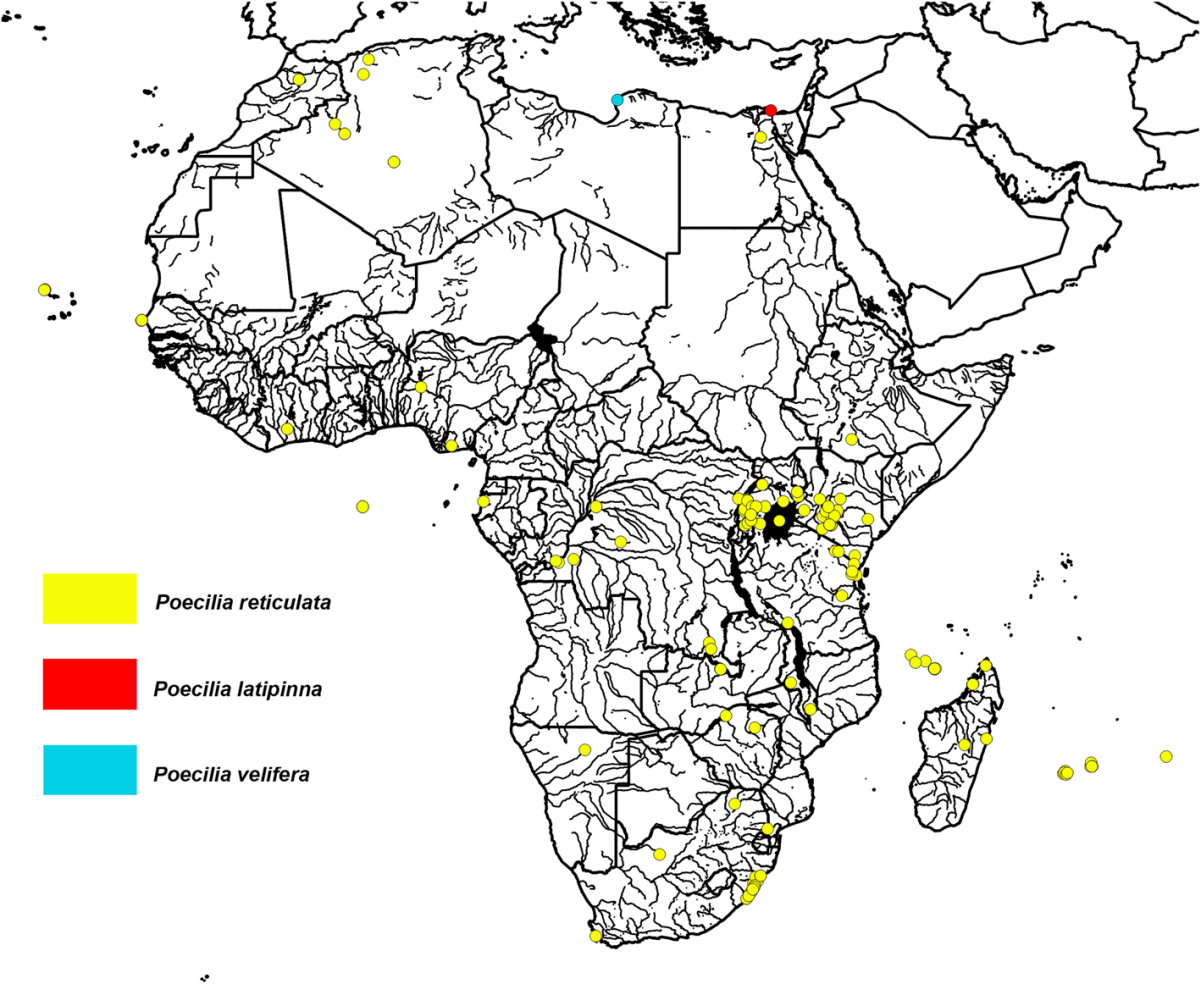
Distribution map of the reported locations for *P. reticulata*, *P. latipinna*, and *P. velifera* in African freshwater systems. All underlying data and sources reported in S1

*Poecilia reticulata* were reported in 41% of African countries (Fig. 4; S1), although there are limited studies of abundant populations [65, 77, 109]. *Poecilia reticulata* occurrences are rare in Northern Africa and was only sampled in Algeria and Morocco in the 1970s [170]. They are considered present in Moroccan freshwaters in the 21st century [46]. Reports from Western Africa and Middle Africa are similarly limited, where *P. reticulata* was introduced to Ghana and the Republic of Congo for mosquito biocontrol, and reported in Cape Verde, and the Democratic Republic of Congo in the 21st Century [94, 114, 170]; S1. Abundant populations were also sampled in sewers in Lagos, Nigeria [109].

In Eastern Africa, *P. reticulata* have been introduced to 11 different countries (Fig. 4; S1). In the 1940s and 1950s, *P. reticulata* was introduced to Kenya, the Mascarene Islands, and Uganda for mosquito biocontrol [45, 111, 186]. They were also introduced to the Comoro Islands in the 1980s for mosquito biocontrol experiments [157] and to Malawi through a private institution, but the latter population did not establish [66]. In the 21st century, *P. reticulata* have been reported in a few locations in Tanzania, Malawi, Rwanda, and the Comoro Islands [34, 45, 77, 154]; while widespread established populations have been sampled in the Mascarene Islands, Kenya, and Uganda [48, 132, 154] (Fig. 4; S1).

*Poecilia reticulata* were first introduced to South Africa from Barbados but these individuals failed to establish [186]. In the late 1980s, introductions associated with the ornamental trade and floods in 1987 leading to the escape of captive individuals and reports of widespread occurrences in Kwa-Zulu Natal [34, 44, 99]. Established *Poecilia reticulata* populations are mostly restricted to urban freshwater habitats in South Africa [143].

### *Poecilia* spp.

**Fig. 5:**
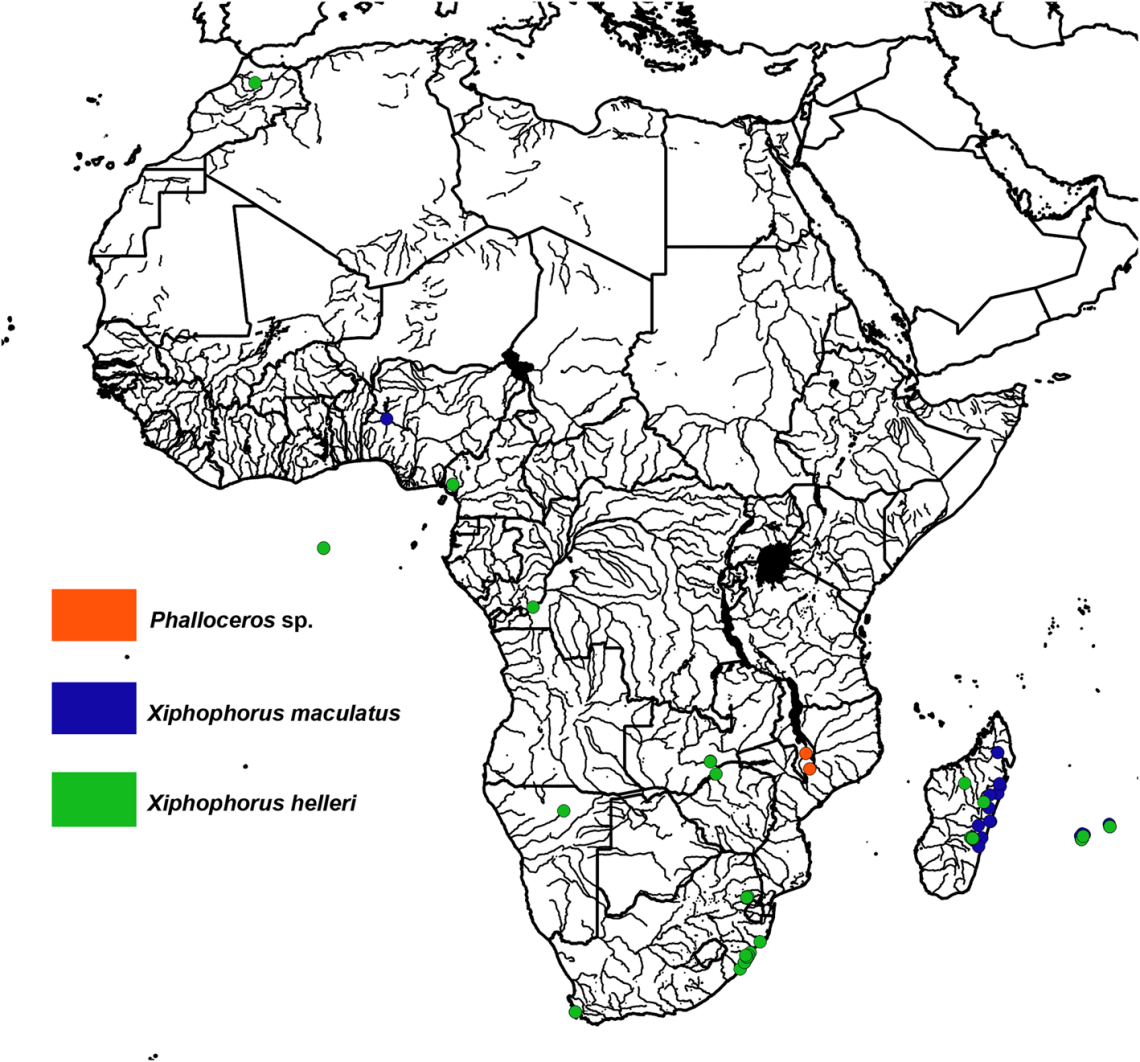
Distribution map of the reported locations for *Phalloceros* sp., *Xiphophorus maculatus*, and *Xiphophorus helleri* in African freshwater systems

In Northern Africa, a range of *Poecilia* spp, were recently sampled in lakes, drains, and a lagoon. *Poecilia latipinna* was collected in Egypt [55], *P. sphenops* in Algeria [76]) and *P*. *velifera* in Libya [57] (Fig. 5; S1).

### *Phalloceros sp.*

A *Phalloceros* sp. was sampled in rivers in Malawi in the 1970s [98] and established populations were reported in 1985 and 1995 (Fig. 5; S1). At the time of sampling the genus only comprised one species, *Phalloceros caudimaculatus*. However, 21 *Phalloceros* spp. are now considered as valid, so identification and confirmation of the specimens from Malawi still needs to be verified.

### *Xiphophorus* spp.

*Xiphophorus* spp. were reported in 24% of African countries. *Xiphophorus hellerii* were first introduced to Africa to Madagascar in the 1950s [72] and later introductions associated with the ornamental trade were to South Africa from Mexico in the 1970s and the Mascarene Islands [44, 102] (Fig. 5; S1). Naturalised *X. hellerii* have been consistently sampled at freshwater sites in KwaZulu-Natal since their introduction [34] but their distribution is localised and are therefore considered as established but not invasive [59]. Established populations were sampled in Madagascar and the Mascarene Islands in the last 20 years [154, 182]. Furthermore, *Xiphophorus hellerii* were sampled in a small number of natural freshwater habitats in the Democratic Republic of Congo, Morocco, and Cameroon [93, 116, 170] (Fig. 5; S1).

*Xiphophorus maculatus* is less commonly found in African countries. The only known introductions of *X. maculatus* were to Nigeria in the 1970s for aquaculture [107] and to South Africa via the ornamental aquarium trade [59]. *Xiphophorus maculatus* was sampled in rivers in the Mascarene Islands in the 1970s [71] and at widespread freshwater sites in Madagascar in the late 1980s and 1990s [151, 172]. Naturalised *X. maculatu*s have been sampled occasionally in Madagascar, the Mascarene Islands, South Africa, and Zambia in the last 20 years [34, 154]) (Fig. 5; S1).

### Impacts

**Fig. 6:**
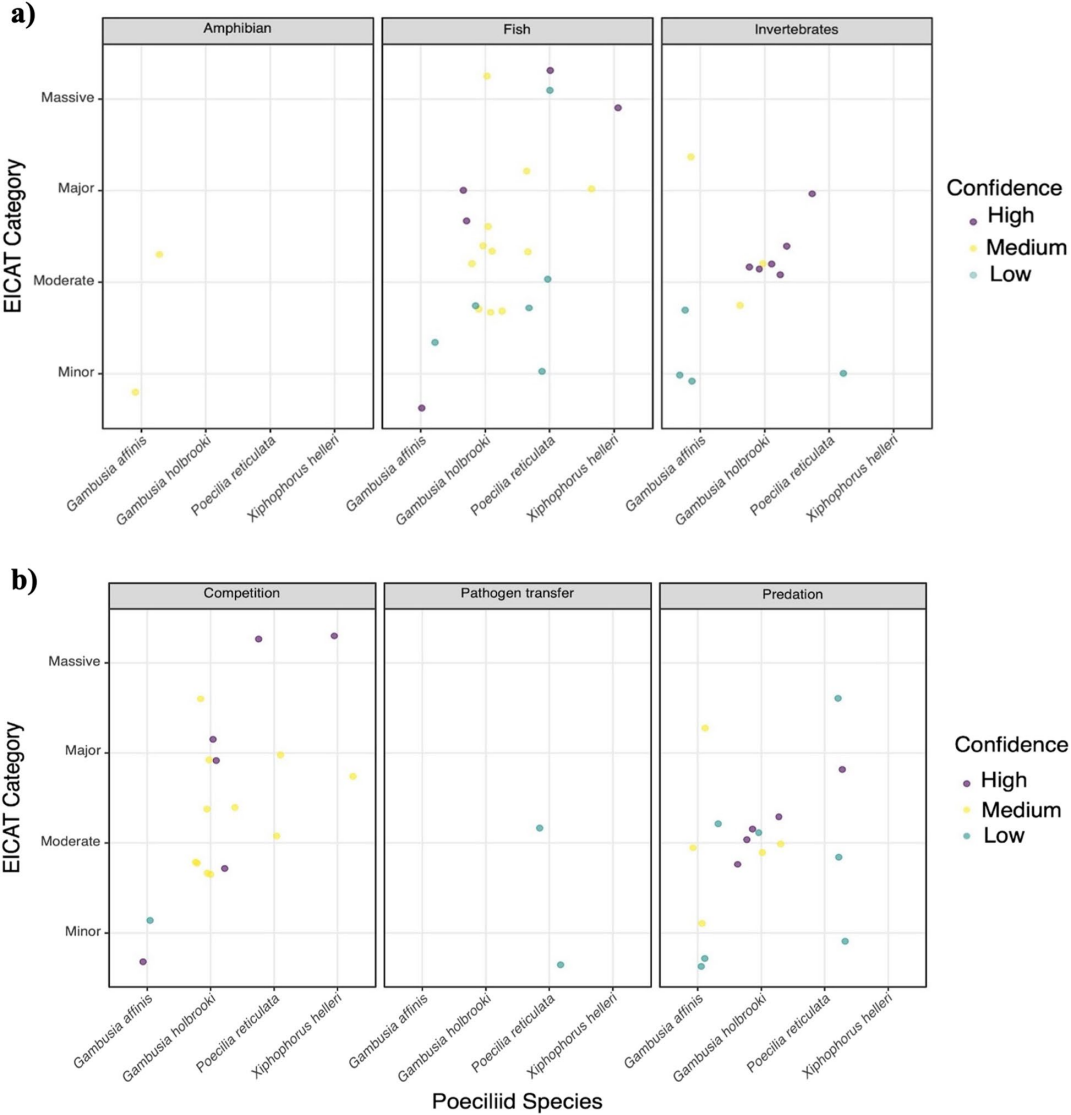
**a** Summary of recorded ecological impacts of invasive poeciliid species on multiple taxa in Africa according to EICAT categories coloured by confidence, **b** Summary of recorded mechanisms of ecological impacts of invasive poeciliid species in Africa according to EICAT categories coloured by confidence. Data sources and justifications for EICAT and confidence scorings found in **S2** and detailed impact evidence in **S3**

We retrieved a total of 37 records of ecological impacts of invasive poeciliids in Africa attributed to *X. maculatus*, *X. helleri*, *P. reticulata* and *Gambusia* spp. Impacts recorded on native fish (*n* = 22, 59.4%), invertebrates (*n* = 13, 35.1%), and amphibians (*n* = 2, 5.4%) through competition (*n* = 18, 48.6%), pathogen transfer (*n* = 2, 5.4%), and predation (*n* = 17, 45.9%) mechanisms (Fig. 6a, b; S2, S3). The confidence in the evidence for the EICAT categories varied with 27% classed as low, 43.2% as medium and 29.7% as high (Fig. 6a, b; S2, S3).

### Mosquitos as a biocontrol agent

Mesocosm field studies were conducted between 1987 and 1988 to evaluate the effectiveness of *P. reticulata* as a vector mosquito biocontrol agent in Grande Comore, Comoro Islands [157]. The percentage of egg rafts for mosquitos decreased from 41 to 6% when exposed to an initial 3–5 *P. reticulata* individuals. This decrease led to a reduction in the infection rate of *Plasmodium falciparum* (parasite causing malaria) in children aged between 5 and 9 years old [157].

When experimentally exposed to *G. affinis*, *Anopheles gambiae* spp. laid less eggs, which was suggested to be a result of kairomone interactions between the two species [33]. Mesocosm experiments in Kenya found *G. affinis* to be the most efficient predator of mosquito larvae out of five natural predators [106], demonstrating the efficiency of mosquitofish as biocontrol agent in Kenya over alternative aquatic predators. However, *G. affinis* was the only fish out of the five predators tested but crucially, no native fish species were tested.

There are anecdotal reports of effective mosquito biocontrol by *Gambusia* spp. throughout Africa, although these are not backed up by empirical evidence. Local experts in Ghana believe introduced *Gambusia* individuals to act as effective mosquito biocontrol agents [46]. In Algeria, *Gambusia* introductions and establishment are thought to have caused declines in the population of the mosquito *Anopheles labranchiae* larvae at Guelma and their eradication at Ouargla [111]. The 1,000 *Gambusia* individuals introduced to Lake Tana, Ethiopia, have been described as initially effective in controlling mosquitoes [175] whilst in Nigeria, *P. reticulata* are considered effective in controlling mosquitoes in Lagos sewers [46, 109].

*Gambusia* spp. have been shown to selectively feed upon vertebrate prey in temporary pond manipulations [83] and long-term monitoring of Algerian freshwater habitats (in Dakhla, Estah, Saulaie and Lac Bleu) [10]. Thus, mosquito larvae are unlikely to be the preferred choice of prey in introduced ecosystems. Furthermore, the vulnerability of intermediate mosquito predators, like *Lovenula raynerae* [39], to *Gambusia* spp. may even cause an increase in mosquito populations following introduction for biocontrol. *Gambusia* spp. introduced for mosquito biocontrol have been ineffective in Uganda [46, 111, 187]. They are also understood to prefer fish larvae over mosquito larvae in Madagascar [151].

## Discussion

Despite being globally invasive, and well documented regarding distribution and ecological impacts across Europe and Australia, there is comparatively limited understanding of invasive poeciliids in Asia and Africa [97, 101, 112, 117]. Lack of information availability and synthesis regarding biological invasions is a barrier to effective policy and legislation formulation, which further exacerbates the freshwater biodiversity crisis [36, 166]. This review summarises and synthesises the state of research on the African continent and confirms the presence and establishment of nine non-native poeciliid species in African freshwaters. There are major data deficits in our knowledge of invasive population status, ecological and economic impact, as well as patchy spread of occurrence records.

### Pathways

The majority of poeciliid introductions at the beginning of the 20th century were directed to vector mosquito biocontrol [186]. Declines in biocontrol-related introduction events followed the discovery of the insecticidal qualities of certain compounds, such as Dichlorodiphenyltrichloroethane (DDT) in 1939 [185]. However, limited pesticide availability and high operational costs renewed interest in larvivorous fish [187], which could explain vector mosquito biocontrol using poeciliids in developing African countries during the 1980s [46, 157]. Other contributing factors likely included pesticide bans by the Stockholm Convention [16], as well as the recognition of their impacts on non-target organisms and persistence in the environment [68, 142, 179]. The complexity of vector mosquito biocontrol is now recognised and must be conducted with a comprehension of potential impacts on ecological processes and interactions [31]. Furthermore, poeciliids used for vector mosquito biocontrol are now known to negatively impact native biota [56, 147]; indigenous species have been demonstrated as efficient control agents [110, 131]; and the use of eco-friendly larvicides is gaining momentum (e.g., “green nanoparticles”) [9]. Therefore, future introductions via this pathway are unadvisable.

The ornamental aquarium trade is relatively unregulated and ubiquitous globally [59, 140] and regulation of the trade is notoriously difficult [124, 149]. Live-bearing ornamental fish, such as poeciliids, are traded more frequently due to their bright colours and higher reproductive success in captivity [11]. Therefore, ornamental trade represents a persistent introduction pathway which has accelerated for poeciliid introductions in Africa since the 1950’s. Challenges in managing and assessing the risk of the ornamental fish introductions arise from difficulties in correctly identifying species in the aquarium trade [121, 181] and insufficient data on the ecological or socio-economic impacts of invasive species required to conduct Socio-Economic Impact Classification of Alien Taxa (SEICAT) assessments [8] and cost-benefit analyses [191]. Field impacts need to be documented urgently to provide evidence for future biosecurity policies.

Genetic barcoding studies of traded taxa in South Africa indicate progress in the field [181] and current DNA barcoding techniques will continue to allow more accurate identification and monitoring of freshwater fish, including poeciliids [17, 156, 182]. Establishing a relationship between commercial ornamental market import/export data and occurrence records has proven problematic [53] and needs to be supported by reliable DNA barcode libraries of aquarium stocks (e.g., [91]). A focus on monitoring the ornamental trade is needed and, despite the difficulty in penetrating it on a global scale [30], improved regulation and records will provide information on the movement of invasive species (e.g., [128]). The illegal trade further complicates matters, although the scrutiny of e-commerce may aid understanding invasion risk and propagule pressure [15, 136].

### Adaptations

The physiological tolerance and behaviours of invasive poeciliids pre-adapt them for establishing populations in novel environments. Extreme plasticity and rapid adaptation potential of invasive poeciliids enables them to colonise sub-optimal environments and exploit vacant niche space [97]. Urban freshwaters are at high risk of invasion due to degradation of waterbodies excluding and extirpating more sensitive native species, thus limiting biotic resistance to poeciliids in these instances, as well as general proximity to high density human populations increasing propagule pressure [2, 10, 35, 171, 184]. In Africa, naturalised poeciliids are found to seasonally alter their dietary niche in response to limited prey availability [109] and adjust their diets towards larger invertebrate prey in diverse communities [83]. These behaviours have contributed to the success of poeciliids in other continents, where they are abundant in unproductive sewage systems [130], capitalising on rich invertebrate and amphibian assemblages [152].

Observations of female-biased poeciliid populations in Africa [28, 57], indicate that there could be some sexual selection acting on the population which may contribute to establishment and spread success. Furthermore, reproductive adaptations such as the production of many offspring of large sizes, female sperm storage [118], and male-biased dispersal [37], allow their efficient colonisation and persistence from small propagule pressure. Invasive poeciliids have successfully established and persisted in freshwater ecosystems despite invading with low propagule pressure [146] and also undergoing severe reductions in genetic diversity [112]. Thus, the pre-adaptations of invasive poeciliids enable their persistence in introduced systems and potential impact on native biodiversity.

### Biodiversity threat

Invasive poeciliids are threatening the conservation status of many African freshwater species and are likely a strong contributing factor in regional declines in biodiversity. For instance, poeciliids have contributed to the decline, exclusion and extirpation of native Aphaniidae and Procatopodidae through competition [46, 74, 162, 184]. Many of these species are facing anthropogenic pressures [50, 182], are classified as Critically Endangered [119] and are trigger species in key biodiversity areas [133]. Invasive poeciliid predation can contribute to population declines in native African invertebrates (e.g. [83], [10]) and amphibians [35, 105]. The invertebrate and amphibian of certain African assemblages are species rich and biodiverse [4, 62, 158], so a regional elimination of taxa is possible and requires urgent empirical assessments to implement mitigation measures. Any alteration in community structure has the potential to cause negative effects and cascading impacts on native populations. Poeciliids contribute to top-down effects via the size-selective predation of invertebrates and overall reduction in organic matter decomposition rates [88]; whereas elsewhere they disrupt trophic cascades by preying upon insectivorous killifish, indirectly increasing decomposition rates [167]. Poeciliids have also been shown to simultaneously drive top-down and bottom-up processes by predation of amphibians, which restructures amphibian assemblages via competitive release, and zooplankton, driving phytoplankton increases [145]. Native species with generalist life-history strategies are found to co-exist with poeciliids [178], whilst specialist and functionally analogous species (e.g. lampeye killifish) are less resistant to invasion [117], which is concerning for the many endemic and geographically restricted species facing declines in African freshwaters. Therefore, biotic homogenisation is a likely outcome of poeciliid invasions, which reduces both taxonomic and functional biodiversity, and will be exacerbated by multiple stressors of climate change and degradation [40].

The current evidence base is relatively sparse considering the long history of poeciliid introductions in the region. There is high confidence evidence of negative poeciliid impacts on invertebrate communities [10, 83] and native killifish [133, 184] which indicates an urgent need for research into negative effects on native biota. Whilst mesocosm tests are useful in analysing direct interactions between species [39, 125, 127], field studies assessing spatial and temporal changes in species composition [137, 138, 169] and the ecological niches of multiple species [126] will provide the high confidence, robust data necessary to inform policy decisions.

#### Climate change and future perspectives

Climate change is influencing the likelihood of invasion and establishment of non-native freshwater species; a trend set to continue in the future [161, 182]. Given that invasive poeciliids inhabit many sub-optimal habitats in Africa [77, 109, 185], dominate hypoxic and brackish ecosystems over a wide distribution [173], and adapt to higher temperatures [97, 126]; deteriorating environmental conditions will continue to favour their establishment. Furthermore, the toxicity of pollutants is likely to increase with rising temperatures [67], which may confer an advantage to tolerant poeciliids over native fauna in urbanised and agricultural freshwaters [58, 65, 173, 185]. The synergism between propagule pressure, climate change and human disturbance can accelerate invasive species establishment and the ensuing ecological impacts [189].

#### Data limitations and recommendations

The recent increase in research on freshwater species compositions in Africa has improved understanding of the geographical distribution of poeciliids [65, 103, 174]. Nonetheless, there are still significant gaps in knowledge, especially outside of Northeast Morocco and South Africa. The scarcity of data means that the threat of invasive poeciliids to native biodiversity is mostly unknown, including the threat to biodiversity hotspots and areas of high conservation value [42, 81]. Additionally, it is extremely difficult to understand and fully quantify the threats represented by invasive poeciliids given the sparse and incomplete systematics of African fish fauna, driven by few local taxonomists, the lack of funding and conflict zones. The taxonomic impediment of the region requires urgent attention as threats caused by invasive species can be underestimated given that information on native species diversity and distribution is superficial [22, 47, 60, 122, 168]. Furthermore, previous ecological research in Africa is biased towards flagship charismatic species, which are not representative of the overall health of freshwater ecosystems [41].

Embracing modern molecular techniques (e.g., DNA barcoding, Single Nucleotide Polymorphism analysis) and ensuring equitable access to these is key to improving capacity to detect and manage invasive poeciliids [168]. A widespread use of cheap eDNA-based monitoring has the potential to remove some barriers to ecological surveys, but this relies on suitable barcoding libraries and a drastic reduction in analysis costs before it can be implemented in an acceptable manner. Intercontinental and intracontinental collaboration which follows knowledge equity principles is strongly recommended and needed to enhance capacity for African ichthyologists [168].

In locations where data is insufficient, invasive species risk assessments will benefit from bioclimatic modelling [97, 129]. Whilst species distribution modelling can inform management strategies [155], but ought to include anthropogenic, environmental, and biotic parameters [20, 89]. Furthermore, citizen science has proven to be a valuable tool in understanding water pollution in African countries [6], so conducting similar surveys with relevant stakeholders and vectors e.g., pet shop owners [181], fish hobbyists [144], and anglers, as well as utilising iNaturalist records may improve knowledge of poeciliid distributions.

## Conclusion

African freshwater ecosystems host rich biodiversity and high levels of endemism which desperately need to be managed and conserved to support biological communities, ecosystem functioning and cultural biodiversity [42, 81]. From our results, it indicates that the threat of non-native poecillids has been underestimated and the biocontrol aspect somewhat over-estimated, hence the continued proliferation of species across waterbodies. We urge for more field evidence of the ecological impact of poecillids on native species to increase management and biosecurity imperatives. Attempts to control invasive poeciliids have been limited in Africa, so future research should be directed at informing management strategies to mitigate their adverse ecological impacts by preventing introduction and removing established populations from isolated waterbodies. However, the plastic nature of poeciliids complicates control strategies and ecosystem approaches are unlikely to be successful in eradication [21]. Restrictions on ornamental importations are minimal [59], so control efforts must focus on educating buyers and limiting their release. Educational campaigns should be directed at multiple stakeholders [19] (e.g., governments, fish hobbyists, fishermen) with a focus on citizens living near vulnerable freshwater habitats. Encouraging people to engage with citizen science and data collection through the iNaturalist platform and other similar initiatives can be a route to rapidly increasing locality knowledge. This can only be achieved through supporting regionally led scientific research, which must be driven by the development of the African research infrastructure, with a focus on current centres of excellence [1, 168] and provision of financial and technological capacity.

## Supplementary Information

Supplementary Material 1.

Supplementary Material 2.

Supplementary Material 3.

## Data Availability

All underlying data is available in the supplementary materials.
