# Supplementary material for: A systematic review of poeciliid fish invasions in Africa
Source: BMC Ecol Evol. 2024 Nov 6;24:136. doi: 10.1186/s12862-024-02321-3 (PMC11539733; doi:10.1186/s12862-024-02321-3)
Supplement: Supplementary file 3 — Supplementary Material 3. [file 12862_2024_2321_MOESM3_ESM.docx]

***Supplementary Material 3 - Detailed description of ecological impact of invasive non-native poeciliids in Africa***

**A systematic review of poeciliid fish invasions in Africa**

Joshua Pritchard Cairns^1^, Pedro Henrique Negreiros de Bragança^2,3^, Josie South^1,2^*

^1^ Water@Leeds, School of Biology, Faculty of Biological Sciences, University of Leeds, Leeds LS2 9JT, UK.

^2^ South African Institute for Aquatic Biodiversity, Private Bag 1015, Makhanda, 6140, Eastern Cape, South Africa.

^3^Department of Ichthyology, American Museum of Natural History, 10024-5102, New York, NY,USA

*Corresponding author: [j.south@leeds.ac.uk](mailto:j.south@leeds.ac.uk)

*Competition*

In Kenya, freshwater populations of the Baringo lampeye (*Lacustricola* sp.), a little-known undescribed killifish which is thought to be critically endangered (pers comms – P. Bragança), are declining most likely due to competition with introduced *P. reticulata* in Lake Baringo (Seegers et al., 2003). In Lake Naivasha, invasive *P. reticulata* is thought to have contributed to the extinction of the Naivasha lampeye (*Micropanchax antinorii*) by outcompeting mature specimens (Seegers et al., 2003). The Naivasha lampeye has not been reported since 1962 (Gherardi et al., 2011). In Morocco, *G. holbrooki* occupy habitats of varying salinities (588 ppm to 1,839 ppm) and oxygen levels (2.01 mg/l to 11.3 mg/l) (Taybi et al., 2020) and potentially exclude the Mediterranean killifish (*Aphanius fasciatus*), which was not recorded even after exhaustive sampling (Taybi, et al., 2020). Furthermore, invasive *G. holbrooki* is understood to have driven population declines of the native Moroccan loach (*Cobitis maroccana*) in Sebou and Loukkos (Deacon, 2011a). As of 2021, *C. maroccana* specimens have now been extirpated from the Sebou River basin (Nogueira et al., 2021). In Algeria, invasive *G. holbrooki* are now abundant where *Aphanius fasciatus* were previously dominant in Lac Bleu (Benslimane et al., 2019). Similarly, competition from *G. holbrooki* has likely excluded the endemic Saharan killifish (*Aphanius saourensis*) from multiple locations (Igli, El Ouata, Kerzaz) in the greater Saoura basin (García et al., 2010). In Lake Oubeira, introduced *G. holbrooki* is thought to have contributed to the reduction in zooplankton populations and competitive exclusion of native *A. fasciatus* and the non-native Twait shad (*Alosa fallax*) (García et al., 2010).

*Gambusia* spp. introductions in Madagascar are likely to be coinciding with the declines of several native fish. *Gambusia* spp. competition is thought to be a driving factor in population declines of the native and endangered Madagascar Panchax (*Pachyanchax sakaramyi*) and contributed to the extinction of *Pantanadon madagascariensis* (Deacon, 2011a). In Manampatrana and Sambava rivers invasive Gambusia spp. are understood to have contributed to the decline of *Bedotia* spp. (Deacon, 2011a); while in Lake Aloatra and Malagasy highlands *Gambusia* spp. introductions are coinciding with decreased numbers of the endemic *Ratsirakia legendrei* (Eleotridae), as well as *Paratilapia* spp. (Deacon, 2011a). Furthermore, native killifish were not detected in eDNA surveys at sites with introduced *G. holbrooki* and *X. maculatus* (Vences et al., 2022). From a total of thirteen sites, native killifish co-exist with introduced poeciliids at only three (*Pachypanchax arnoulti* with *X. hellerii* at one, and *Pachypanchax omalonotus* with *P. reticulata* at two (Vences, et al., 2022)). *Pachypanchax arnoulti* was once abundant in streams near Maevatanana, in the Betsiboka-Ikopa drainage, however, it is now absent there and invasive *X. hellerii* is dominant (Loiselle, 2006). Moreover, *P. arnoulti* is under threat from invasive *X. hellerii* in the Ampijoroa Forest Reserve and *G. holbrooki* in the Kamoro River (Loiselle, 2006).

In South Africa, *G. affinis* outcompetes the native River Goby (*Glossogobius callidus*) by reducing River Goby predation rate (Mofu et al., 2019b). Stable isotope analysis in Sundays River Valley irrigation ponds finds *G. affinis* to exhibit a broad dietary niche, overlapping considerably with the native Mozambique tilapia (*Oreochromis mossambicus*) (Mofu et al., 2023). Negative spatial interactions arise from resource competition between *G. affinis* and *G. callidus* (J. South – unpublished data; Mofu et al., 2023). However, the varying diets of *G. affinis* size classes indicate greater predator efficiency and prey-switching throughout the water column, potentially in an attempt to avoid competition with River Goby (Mofu et al., 2023).

*Predation*

Invasive poeciliids have been reported to prey upon smaller fry of indigenous killifish. *Poecilia reticulata* is understood to have contributed to the extirpation of native *Micropanchax antinorii* in Lake Naivasha, Kenya, by feeding on juveniles (Seegers et al., 2003). In Madagascar, *Pachypanchax sakaramyi* declines are considered to be a result of high predation pressure on juvenile fish by *G. holbrooki* and *P. reticulata* (Loiselle, 2006).

Introduced *G. affinis* is potentially driving population declines in Gray’s stream frog, *Strongylopus grayii,* and Delalande’s sand frog, *Tomopterna delalandii* (Conradie, 2018). When exposed to *G. affinis*, *S. grayii* were the preferred prey (91.7% consumed within a week of introduction), while *T. delalandii* exhibited stunted tadpole growth and higher levels of risk-reduction activity (Conradie, 2018). In Potchefstroom ponds in South Africa, increasing abundance of *G. affinis* predators is positively correlated with amphibian species richness (Kruger et al., 2015). Predation of more dominant species in the ecosystem is suggested to facilitate the success of many diminutive species, and therefore allowing a more diverse amphibian community (Kruger et al., 2015). *Gambusia affinis* was the most common predator detected in the study (44% of sites), however, the combined impacts of multiple predators were assessed (Kruger et al., 2015).

Comparative functional response and stable isotope analysis in the Sundays River Valley also in South Africa found *G. affinis* to exert a higher per capita ecological impact upon lower trophic levels than native *Glossogobius callidus* at two different temperature treatments (18°C and 25°C) (Mofu et al., 2019a). Abundant summer *G. affinis* populations in ML swart irrigation pond, Sundays River Valley, also exhibit greater impact potential (Mofu et al. 2019b), so there is inferred high predation on chironomid larvae from introduced *G. affinis*. However, due to reduction in native species predation as a result of competition, there may be a net neutral effect of predation on prey populations (Mofu et al. 2019b). Calanoid copepod *L. raynerae* appears naïve to *G. affinis* olfactory and visual cues which may make populations at risk from high predation (Cuthbert et al. 2018).

A manipulative approach into the impact of invasive *G. holbrooki* on zooplankton communities within temporary ponds in El Feid, Algeria, found mosquitofish to exhibit size-selective predation of invertebrate prey (Haiahem et al., 2018). *Gambusia holbrooki* preferred larger individuals from the families Anostraca, Daphniidae, and Ostracoda, and quickly eradicated these populations. Smaller zooplankton (Cyclopoid copepods, Cladocera, smaller Ostracoda) population abundances peaked in the initial period as *G. holbrooki* preferentially preyed on larger invertebrates, but once these resources were used up, *G. holbrooki* switched to feeding on the smaller invertebrates and eliminated their populations (Haiahem et al., 2018).

Monitoring of dune ponds in north-eastern Algeria between 1996 and 2013 found Notonectidae species abundance to decline significantly in the presence of *G. holbrooki* (Benslimane et al., 2019). *Gambusia holbrooki* also indirectly impacted Notonectidae via consumption of their primary food source, zooplankton (Benslimane et al., 2019). Corixidae species did not decline in the presence of mosquitofish overall, but population declines were apparent in the water boatman *Hesperocorixa moesta*. Furthermore, *Gerris throracicus* (water skater) and *Mesovelia vitigera* (water treader) were eradicated towards the end of the study, indicating a feeding preference by *G. holbrooki* on surface-dwelling invertebrates, explaining the heightened survival rate of benthic corixids (Benslimane et al., 2019).

In Lake Nkuruba in Uganda, assessment of mercury biomagnification finds invasive *P. reticulata* to dominate the ecosystem and feed on benthic invertebrates and plant detritus (Campbell et al., 2006), indicating pressure upon invertebrate communities from introduced specimens.

*Co-introduced parasites*

In South Africa, the presence of an invasive nematode *Camallanus cotti*, originally from Asia, has been identified on introduced *P. reticulata* individuals (Tavakol et al., 2017). Both captive and naturally occurring *P. reticulata* specimens have been identified as the host of *C. cotti* parasite. Similarly, *Ichthyophthirius multifiliis*, an external parasite, has been co-introduced with ornamental *P. reticulata* as it was revealed by an investigation on commercial aquarium fish suppliers in South Africa (Mouton et al., 2001), which is understood to have driven declines in native freshwater species (Ellender and Weyl, 2014).
